# Shape Memory Polymer Foams with Tunable Degradation Profiles

**DOI:** 10.1021/acsabm.1c00516

**Published:** 2021-08-11

**Authors:** Anand Utpal Vakil, Natalie Marie Petryk, Ellen Shepherd, Henry T. Beaman, Priya S. Ganesh, Katheryn S. Dong, Mary Beth B. Monroe

**Affiliations:** Department of Biomedical and Chemical Engineering, Syracuse Biomaterials Institute, and BioInspired Syracuse: Institute for Material and Living Systems, Syracuse University, Syracuse, New York 13244, United States

**Keywords:** shape memory polymers, polyurethanes, oxidation, degradation, foams

## Abstract

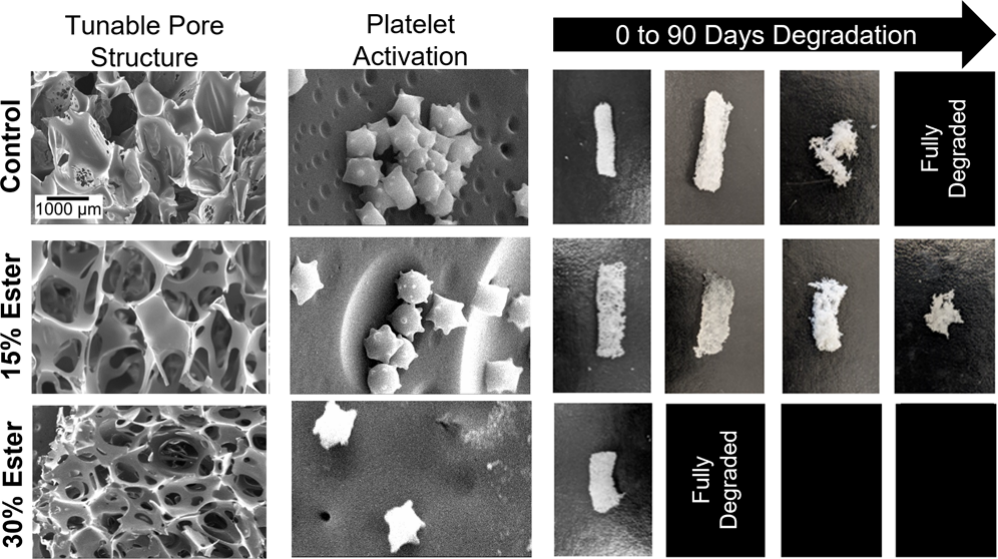

Uncontrolled hemorrhage is the leading cause of preventable death on the battlefield and results in ∼1.5 million deaths each year. The primary current treatment options are gauze and/or tourniquets, which are ineffective for up to 80% of wounds. Additionally, most hemostatic materials must be removed from the patient within <12 h, which limits their applicability in remote scenarios and can cause additional bleeding upon removal. Here, degradable shape memory polymer (SMP) foams were synthesized to overcome these limitations. SMP foams were modified with oxidatively labile ether groups and hydrolytically labile ester groups to degrade after implantation. Foam physical, thermal, and shape memory properties were assessed along with cytocompatibility and blood interactions. Degradation profiles were obtained in vitro in oxidative and hydrolytic media (3% H_2_O_2_ (oxidation) and 0.1 M NaOH (hydrolysis) at 37 °C). The resulting foams had tunable, clinically relevant degradation rates, with complete mass loss within 30–60 days. These SMP foams have potential to provide an easy-to-use, shape-filling hemostatic dressing that can be left in place during traumatic wound healing with future potential use in regenerative medicine applications.

## 1 Introduction

Hemorrhage is the leading cause of potentially survivable death on the battlefield. Up to 90% of preventable deaths are due to uncontrolled bleeding, and approximately 20% of combat casualties result in death before the injured can be transported to a treatment facility.^1−3^ The most common hemorrhage treatment includes the use of tourniquets and gauze coated with coagulants. However, gauze is often ineffective at promoting clotting, and improper or prolonged tourniquet use can lead to complications like nerve paralysis, limb ischemia, arrhythmias, and crush syndrome, which can result in amputation above the position of a tourniquet.^4,5^ This urgent clinical need has led to the recent development of new options for hemorrhage control. For example, XStat is designed for bleeding control from junctional wounds.^6^ XStat contains ∼95 oxidized cellulose foam pieces that are injected into the wound using a syringe-like applicator, after which they expand and fill up the wound to apply pressure and induce clotting.^7^ Each foam piece must be removed within 4 h to prevent ischemia, and XStat exhibits a 22-fold increase in removal time compared with gauze.^6,8^ This removal process can also lead to rebleeds of surrounding tissues. Therefore, XStat may not be the best alternative to gauze in remote locations where access to a fixed care facility is limited.

To overcome limitations of current hemostatic dressing options, an ideal hemostatic material is biocompatible, promotes rapid blood clotting, and is degradable. A degradable hemostatic dressing could theoretically be left in place after application to degrade during healing, prolonging the time available to get a patient to a fixed facility and reducing rebleed risks upon removal. To that end, He et al. prepared oxidized regenerated cellulose (ORC) gauze.^9^ In a rabbit liver and ear-artery injury, this material induced hemostasis and degraded fully within 21 days; however, the gauze was forcibly removed by the blood flow from the artery, which reduces its applicability in traumatic hemorrhage control. Dai et al. developed silver-exchanged calcium-doped mesoporous silica spheres for hemorrhage control.^10^ The particles achieved hemostasis within 340 s and underwent a 40% weight loss over 42 days in vitro. However, these mesoporous particles have a recorded pore size of 3.2 nm, which is likely too small to facilitate tissue ingrowth.^11^ Porous chitosan-based hemostatic microparticles were developed by Li et al.^12^ The microspheres exhibit an increase in blood clotting with decreased surface pore size and a 40% weight loss over 4 weeks of lysozyme incubation but have a pore size of <2 μm. Thus, these microparticles may not be suitable for deep wounds to aid tissue ingrowth.

To address this clinical need, shape memory polymer (SMP) foams are being investigated as hemostatic biomaterials.^13^ SMPs are “smart” stimuli-responsive materials that are synthesized in a primary, permanent shape, triggered using an external stimulus, such as heat or light, and strained/fixed into a temporary, secondary shape that is retained upon removal of the external stimulus. After re-exposure to the stimulus, SMPs regain their primary shape.^14^ Heat is used as an external stimulus in this polyurethane SMP foam system, and shape change is designed around the polymer’s glass-transition temperature (*T*_g_). These foams are biocompatible and capable of promoting rapid blood clotting due to their thrombogenic surface chemistry and high surface area.^15,16^

The shape memory properties allow SMP foam radial compression and storage in a low-profile, temporary geometry at temperatures below their *T*_g_. Foam *T*_g_’s are reduced by exposure to water (relative to *T*_g_ in dry conditions) due to plasticization of the polymer network.^17^ Therefore, SMP foams can be stored compressed at relatively high temperatures (∼40 to 50 °C) in the dry state. This smaller volume material can theoretically be packed into deep and/or irregularly shaped wounds, which is particularly important in gunshot wounds, which often have small entry points that expand outwards into large internal wound cavities.^18^ Once exposed to water present in blood at body temperature (37 °C), the foam *T*_g_ is reduced, allowing expansion back to the original shape to fill up the space of wound, clotting the blood and reducing further hemorrhage. One of the main advantages of these foams over commercially available hemostatic materials is that their chemistry can be tuned according to application requirements. The goal of the current work is to modify the SMP foams to degrade after implantation to enable prolonged use and reduce rebleed risks during removal.

Weems et al. found that SMP foams undergo oxidative degradation via scission of tertiary amines in the monomers.^19^ “Real-time” in vitro degradation studies in 3% H_2_O_2_ revealed a 50% mass loss over ∼100 days. To increase the degradation rate, previous efforts focused on incorporating hydrolytically degradable ester linkages into the polymer network. Singhal et al. added poly(caprolactone) macromers into the system.^20^ These foams had a relatively low *T*_g_ around 20 °C, which limits their stable storage in the secondary shape, and mass losses were slow, even in accelerated degradation media.^21^ Weems et al. synthesized succinic acid-based ester-containing foams with higher *T*_g_.^22^ However, degradable formulations still had relatively low dry *T*_g_ (∼37 °C) and mass loss rates that are slower than wound healing rates (complete mass loss in 80 days in 2% H_2_O_2_). To improve upon this system, degradable SMP foams with appropriate thermal properties (*T*_g_ > 50 °C) were developed by Jang et al. using ester-containing trifunctional monomers.^23^ However, the fastest complete mass loss was observed within 90 days in an accelerated oxidative degradation medium (20% H_2_O_2_). Thus, SMP foams with appropriate thermal properties and more rapid degradation rates to better match tissue regeneration are still required.

Due to the hydrophobicity of the foams, we hypothesized that clinically relevant degradation rates (∼6 to 8 weeks based on previous clinical data^24^) could be obtained by increasing the local hydrophilicity around hydrolytically degradable ester groups. To that end, we synthesized new monomers by esterifying nitrilotriacetic acid (NTA) with diethylene glycol (DEG) and incorporated the resulting ester-containing monomer into SMP foams. NTA includes an oxidatively degradable tertiary amine, DEG increases hydrophilicity next to the hydrolytically degradable ester linkages, and the ether linkages of DEG are susceptible to oxidative degradation.^20^ After characterizing scaffold properties, cytocompatibility, and blood interactions, degradation was assessed in 3% H_2_O_2_ at 37 °C to mimic real-time oxidative degradation in the body and in an accelerated hydrolytic degradation solution (0.1 M NaOH).^25,26^ Mass loss, pore size/structure, surface chemistry, and *T*_g_ were measured over time to establish in vitro degradation profiles. These SMPs have the potential to provide an easy-to-use, shape-filling hemostatic dressing that can be left in place during traumatic wound healing.

## 2 Materials and Methods

### 2.1 Materials

Hydrogen peroxide (H_2_O_2_, certified ACS, 30%), sodium hydroxide (NaOH), ethanol (reagent alcohol), chloroform, nitrilotriacetic acid (NTA), 1-(3-dimethylaminopropyl)-3-ethylcarbodimide HCl (EDC), 4-(dimethylamino)pyridine (DMAP, ≥99%), hexamethylene diisocyanate (HDI), N,N,N′,N′-tetrakis-(2-hydroxypropyl)-ethylenediamine (HPED), triethanolamine (TEA), diethylene glycol (DEG), Triton X-100, and phosphate-buffered saline (PBS) were purchased from Fisher Scientific (Waltham, MA) and used as received. Porcine blood was purchased from Lampire Biological Laboratories (Pipersville, PA). Glutaraldehyde was purchased from Electron Microscopy Sciences (Hatfield, PA). EPH-190, BL-22, and T-131 were provided by Evonik (Essen, Germany) and used as received.

### 2.2 Synthesis of Ester-Containing Triol

**Figure 1:**
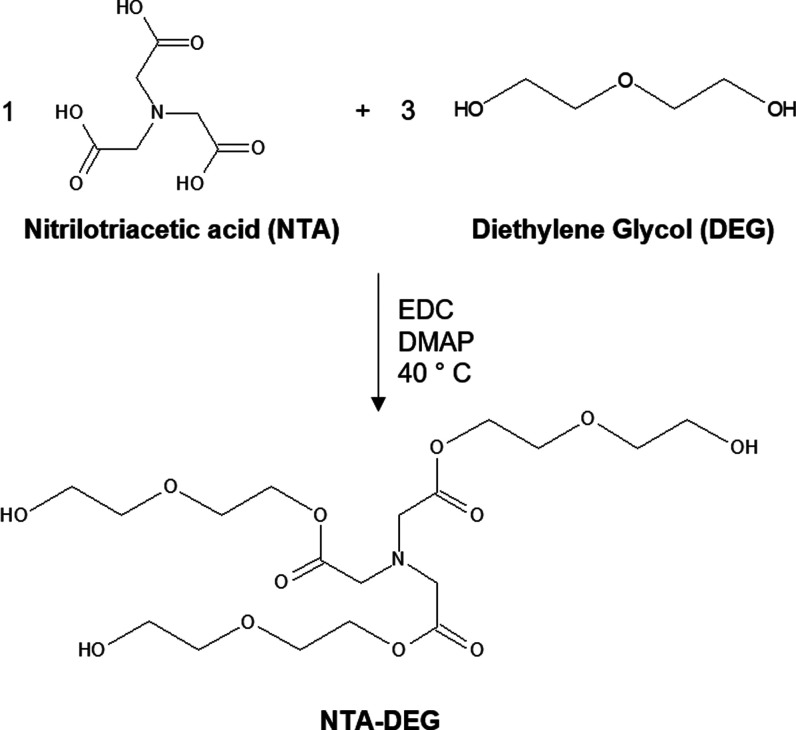
Synthesis of ester-containing monomer, nitrilotriacetic acid (NTA)–diethylene glycol (DEG).

NTA and DEG were added to chloroform at a 1:3 molar ratio with 0.1 mol. equiv of DMAP and 3 mol. equiv of EDC as catalysts (Figure 1). The reaction was carried out at 40 °C in a nitrogen environment over molecular sieves, which were added to capture water produced during the esterification reaction. Attenuated total reflectance Fourier transform infrared spectroscopy (ATR-FTIR, Nicolet iS10, Thermo Scientific) was carried out on the reaction product every 24 h to track its completion according to the introduction of a peak at ∼1714 cm^–1^ that corresponds with the C=O of the ester. Upon reaction completion, excess solvent was vaporized using rotary evaporation, and the final product was dried overnight under vacuum. The dried product, NTA–DEG, was analyzed using ATR-FTIR and ^1^H-nuclear magnetic resonance (NMR, Bruker Avance III HD 400 MHz) spectroscopy to confirm the formation of ester linkages. Successful esterification of DEG was indicated by an ester peak at 1741 cm^–1^ in the FTIR spectra (Figure S1a). NMR spectra were collected in CDCl_3_ at 298 K using the TMS/solvent signal as an internal reference. NTA–DEG: ^1^H NMR (CDCl_3_; ppm): 3.64 (t, −CH_2_CH_2_OCO−), 3.64 (t, −CH_2_OH), 3.72 (s, −CH_2_N−), 3.78 (t, −CH_2_CH_2_OH), and 4.25 (s, −CH_2_OCO−). NMR confirmed 85–88% functionalization of NTA carboxylic acids with DEG (Figure S1b).

### 2.3 Foam Synthesis

Polyurethane foams were fabricated using a two-step process. In the first step, an isocyanate (NCO) premix was prepared that contained 100 mol % of required isocyanates from HDI and a fraction of hydroxyl equivalents from HPED, TEA, and NTA–DEG. The premix was reacted at 50 °C for 48 h. The remaining mol % of hydroxyl components was mixed with catalysts (T-131 and BL-22) and a blowing agent (deionized (DI) water). Surfactant (EPH-190) was added to the premix after the 48 h cure. The NCO premix and hydroxyl components were mixed in a high-speed mixer (Flacktek, Landrum, SC) and poured into a large beaker, which was incubated at 50 °C for 5–10 min to allow for foam formation. Synthesized foam compositions are shown in Table 1.

**Table 1:** Synthesized Foam Compositions

sample ID	HDI (wt %)	HPED (wt %)	TEA (wt %)	DEG (wt %)	NTA–DEG (wt %)	EPH-190 (wt %)	T-131 (wt %)	BL-22 (wt %)	water (wt %)
Control	54.03	27.61	8.05			6.44	0.46	1.01	2.37
15% NTA–DEG	49.45	25.27		3.93	11.23	6.44	1.20	1.01	2.40
30% NTA–DEG	43.10	24.40			21.71	6.17	1.10	1.23	2.29

### 2.4 Foam Pore Analysis

Pore sizes of samples were measured using scanning electron microscopy (SEM). Samples (*n* = 3, 1 cm^2^) were cut along the vertical and lateral foam axes and sputter-coated with gold using a Denton Vacuum sputter coater before imaging (Jeol JSM 5600) at 35× magnification and a 10 kV high vacuum. The micrographs were analyzed using ImageJ software to quantify pore diameters.

### 2.5 Density

Samples (*n* = 3) were cut into cubes (1 cm^3^) using a hot wire cutter, and the length of each face was measured using digital calipers. Measurements were converted to volumes, and samples were then weighed to determine densities.

### 2.6 Mechanical Testing

Samples (*n* = 3) were cut in dog bone shapes (ASTM D638 scaled down by a factor of 4) with a gauge length of 6.25 mm and a width of 1.5 mm. The samples were tested in both dry and wet conditions. To measure wet tensile properties, samples (*n* = 3) were placed in 50 °C DI water for 5 min and pressed dry prior to fixing on the tensile tester. Samples were placed into a tensile tester with a 24 N load cell and stretched at a rate of 2 mm/min until failure to measure elastic modulus, elongation at break, and ultimate tensile strength.

### 2.7 Thermal Analysis

A Q200 differential scanning calorimeter (DSC, TA Instruments, New Castle, DE) was used to measure *T*_g_. Samples (*n* = 3, 3–5 mg) were placed in t-zero aluminum pans and then equilibrated at −40 °C, heated to 120 °C at 10 °C/min, kept isothermally for 2 min, cooled to −40 °C, kept isothermally for 2 min, and heated to 120 °C at 10 °C/min. Dry *T*_g_ was measured as the half-height transition temperature during the second heating cycle. To measure wet *T*_g_, samples (*n* = 3) were placed in 50 °C DI water for 5 min, pressed dry, and placed in t-zero aluminum pans prior to running a single heating cycle.

### 2.8 Swelling Ratio

Cylindrical foam samples (∼20 to 30 mg in dry weight) were cut, cleaned in DI water and 70% ethanol, and dried under vacuum at 40 °C. Their dry masses (*W*_d_) were obtained (*n* = 3), and then they were placed in 37 °C water for 5 min or 24 h. Samples were patted dry on a laboratory wipe and then weighed to obtain swollen masses (*W*_w_). Swelling ratio (SR) was calculated as
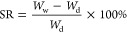


### 2.9 Shape Memory Behavior

Cylindrical foam samples (1 cm length, 8 mm diameter) were cut, cleaned in DI water and 70% ethanol, and dried under vacuum at 40 °C. Cleaned samples were heated to 100 °C for 10 min. The diameter (*n* = 3) was measured using digital calipers prior to crimping cylinders in a radial compression crimper (Blockwise Engineering, Tempe, AZ) and cooling them down while crimped. Crimped samples were placed in vials in a dry box containing a desiccant for 24 h and then fixed on a 330 μm Nitinol wire. Their diameters were measured again, and then they were placed in a DI water bath at 37 °C. Expansion profiles were captured using a camera that recorded images every 5 s for 5 min. The images were processed using Insight Toolkit (ITK) to measure the change in diameter over time. Foam area (number of pixels) from each image was normalized against that of the last image with the known diameter (measured using calipers after foam was removed from the water bath), and % volume recovery was calculated as
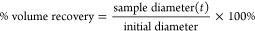
Foam diameter vs time was plotted over the expansion time frame.

### 2.10 Spectroscopic Analysis

Surface chemistry of foams was characterized by collecting ATR-FTIR spectra on thin slices of foam at a 0.8 cm^–1^ resolution.

### 2.11 Cytocompatibility

NIH/3T3 Swiss mouse fibroblasts (ATCC–CCL92) were cultured with Dulbecco’s modified Eagle’s medium (DMEM, high glucose GlutaMAX) supplemented with 10% heat-inactivated fetal bovine serum (FBS) and 1% penicillin–streptomycin (P/S, Gibco) at 37 °C/5% CO_2_. For all studies, cells (between passages 4 and 6) were used after 3 days of culture. Cells were seeded in a 24-well tissue-culture polystyrene plate at 10,000 cells/well and cultured for 24 h. Morphology was assessed using a Zeiss Axiovert inverted microscope to confirm even cell distribution. Media was removed, and cells were washed with sterile phosphate-buffered saline (PBS) prior to exposure to samples. Then, the cleaned foam pieces were placed in Transwell inserts in the preseeded plates along with fresh DMEM with 10% FBS and 1% P/S. Positive (cytocompatible) controls included wells with empty inserts, and negative (cytotoxic) controls included wells with empty inserts and 0.5% H_2_O_2_ in media.

Following incubation over 3, 24, and 72 h, a Resazurin cell viability assay was utilized to quantify cytocompatibility. Transwell inserts and solutions were removed from wells and replaced with the Resazurin cell viability stain for 4 h at 37 °C. Then, a plate reader (FLx800, BioTek Instruments, Inc.) was used to measure absorbance at 570 nm. Cell viability was calculated as
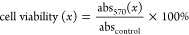
where *x* is the selected treatment group, and the empty insert control is used as a standard that equals 100% viability.

### 2.12 Blood Interactions

Porcine blood (Lampire Biological Laboratories, Pipersville, PA) anticoagulated with Na-citrate upon collection was stored at 4 °C for up to 3 weeks from the bleed date. Control, 15% NTA–DEG, and 30% NTA–DEG foams were washed and dried prior to characterization in all studies. QuikClot Combat Gauze was included as a clinical control. Blood absorption was analyzed by weighing dried samples (*n* = 3; ∼50 mg) and incubating them in blood at 37 °C. Samples were weighed at 24 h, and blood absorption was calculated as
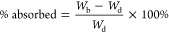
where *W*_b_ is the mass of the sample in blood and *W*_d_ is the dry mass.

Coagulation time was measured by placing samples (*n* = 4) in 1.5 mL tubes, with empty tubes serving as negative (nonclotting) controls. Blood was brought to room temperature, and a 1 M CaCl_2_ solution was added to obtain a final concentration of 0.01 M CaCl_2_ and reverse the anticoagulant. Then, 50 μL of blood was added to each sample tube. At each time point (every 6 min over 30 min), 1 mL of DI water was added to the tubes to stop the clotting process and lyse free red blood cells. Tubes were centrifuged (2300 rpm, 15 min), inverted, and imaged using a digital camera. Then, 200 μL of lysate was pipetted from each tube into a 96-well plate, and absorbance was measured at 540 nm using a BioTek Synergy 2 Multi-Mode Microplate Reader (Winooski, VT) to determine the relative amount of hemoglobin released at each time point.

### 2.13 Platelet Attachment

An LDH cytotoxicity assay kit (Cayman Chemical, Ann Arbor, MI) was used to quantify the attachment of platelets to samples. To obtain a standard curve, whole blood was centrifuged at 3000 rpm for 15 min to obtain platelet-rich plasma (PRP). Multiple concentrations of PRP were prepared by diluting with PBS at 100, 50, 25, 12.5, and 6.5% to generate a standard. Hemocytometer counts at each PRP concentrations (*n* = 4) were acquired and used to quantify standard values.

SMP foams (*n* = 4) were cut to have equal surface areas and placed in the wells of a 24-well plate. Gauze was used as a clinical control. Then, 1 mL of whole blood was added to each well and the plate was incubated at 37 °C for 30 min. Nonattached platelets were washed away with PBS. Samples were transferred to another plate containing 1 mL of fresh PBS and 100 μL of 10% Triton X-100 and incubated at 37 °C for 1 h to lyse the attached platelets. Then, 100 μL of supernatant was taken from each sample well and transferred to a 96-well plate. The LDH reaction solution (100 μL) was added to each well, and the plate was incubated for 30 min at 37 °C on an orbital shaker. Following incubation, absorbances were read on the microplate reader at 490 nm.

### 2.14 Platelet Activation

SMP foams (approximately 0.5 cm^3^) were incubated in whole blood and rinsed of nonattached platelets. To observe activity states and activation of the attached platelets, samples were prepared for SEM imaging. Samples were fixed in a solution of 2% glutaraldehyde (Electron Microscopy Sciences, Hatfield, PA) overnight at 4 °C. Following fixation, samples were dehydrated in solutions with increasing concentrations of ethanol: (1) 30 min in 50% ethanol, (2) 30 min in 70% ethanol, (3) 30 min in 95% ethanol, and (4) 30 min in 100% ethanol. The final dehydration was accomplished through drying overnight in a vacuum oven at room temperature, and samples were sputter-coated with 5–10 nm of gold. SEM analysis was performed using a Jeol NeoSCope JCM-5000 scanning electron microscope at an operating voltage of 10 kV. Random regions of interest were imaged at 1000× and 5000× magnifications. Images were analyzed qualitatively for signs of platelet aggregation and morphology change.

### 2.15 Degradation Analysis

Cylindrical foams (*n* = 8, 8 mm diameter, 1 cm height) were washed and dried, and initial masses were obtained using a gravimetric scale. Samples were placed in 3% H_2_O_2_ (real-time oxidative degradation media) or in 0.1 M NaOH (accelerated hydrolytic degradation media) at 37 °C. Every 3 days, the degradation media was changed. At selected time points, samples were washed with ethanol and dried under vacuum for 24 h. After drying, samples were imaged using a camera, and masses were measured (*n* = 5). A thin slice was cut from a sacrificial set of foams (*n* = 3) and used to measure pore morphology, *T*_g_, and surface chemistry as described above.

### 2.16 Statistical Analysis

Measurements are presented as mean ± standard deviations. Student’s *t* tests were performed to determine differences between NTA–DEG foams and controls. Statistical significance was taken as *p* < 0.05.

## 3 Results

### 3.1 Structural Properties

**Figure 2:**
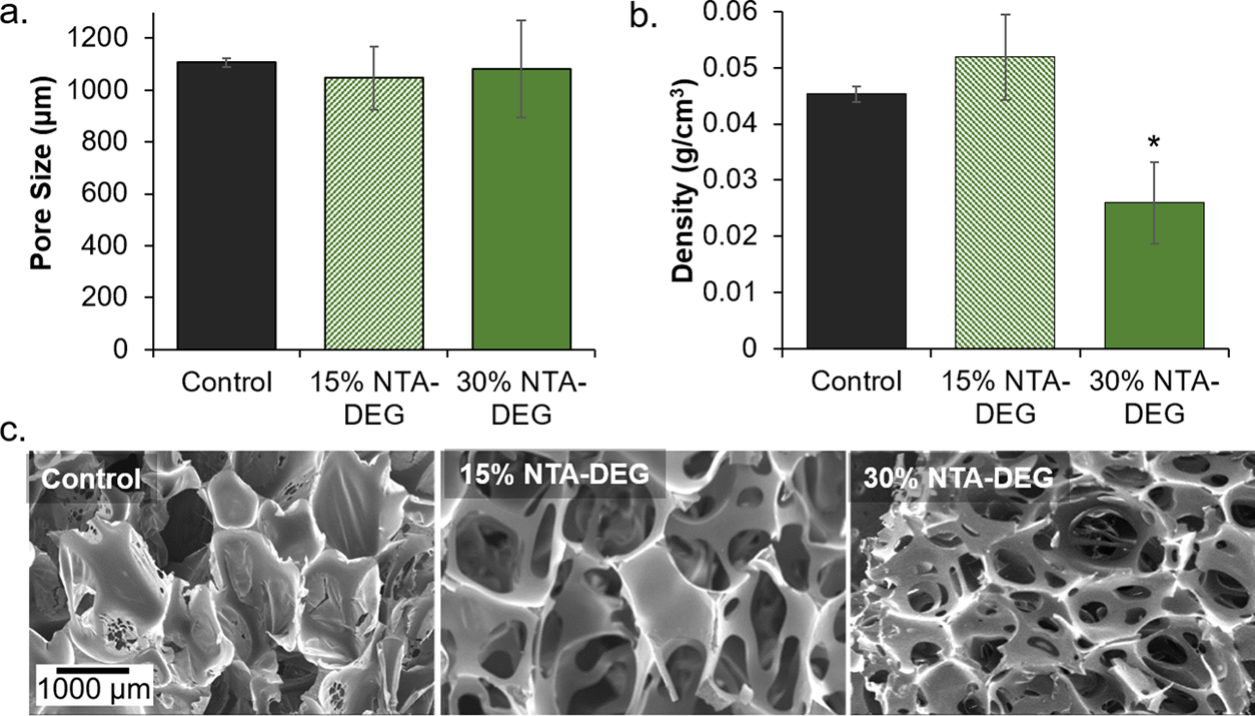
Structural properties of synthesized foams. (a) Average pore size of foams (*n* = 6) measured using SEM micrographs and subsequent SEM analysis. (b) Foam densities (*n* = 3). (c) Representative SEM micrographs of foam samples used for pore analysis. Scale bar of 1000 μm applies to all images. Average ± standard deviation displayed in panels (a) and (b). *p < 0.05 relative to control foam.

All formulations had a pore size of approximately 1100 μm, and both NTA–DEG formulations had comparable pore sizes to the control (Figure 2). In general, foam densities were low (<0.06 g/cm^3^) for all formulations, demonstrating that the NTA–DEG monomer can be incorporated into low-density foams. The addition of NTA–DEG resulted in pore opening, with interconnects visible in pore walls (Figure 2c), which corresponds with a significantly reduced density in the 30% NTA–DEG foams relative to that of the control foam.

### 3.2 Thermal Properties

**Figure 3:**
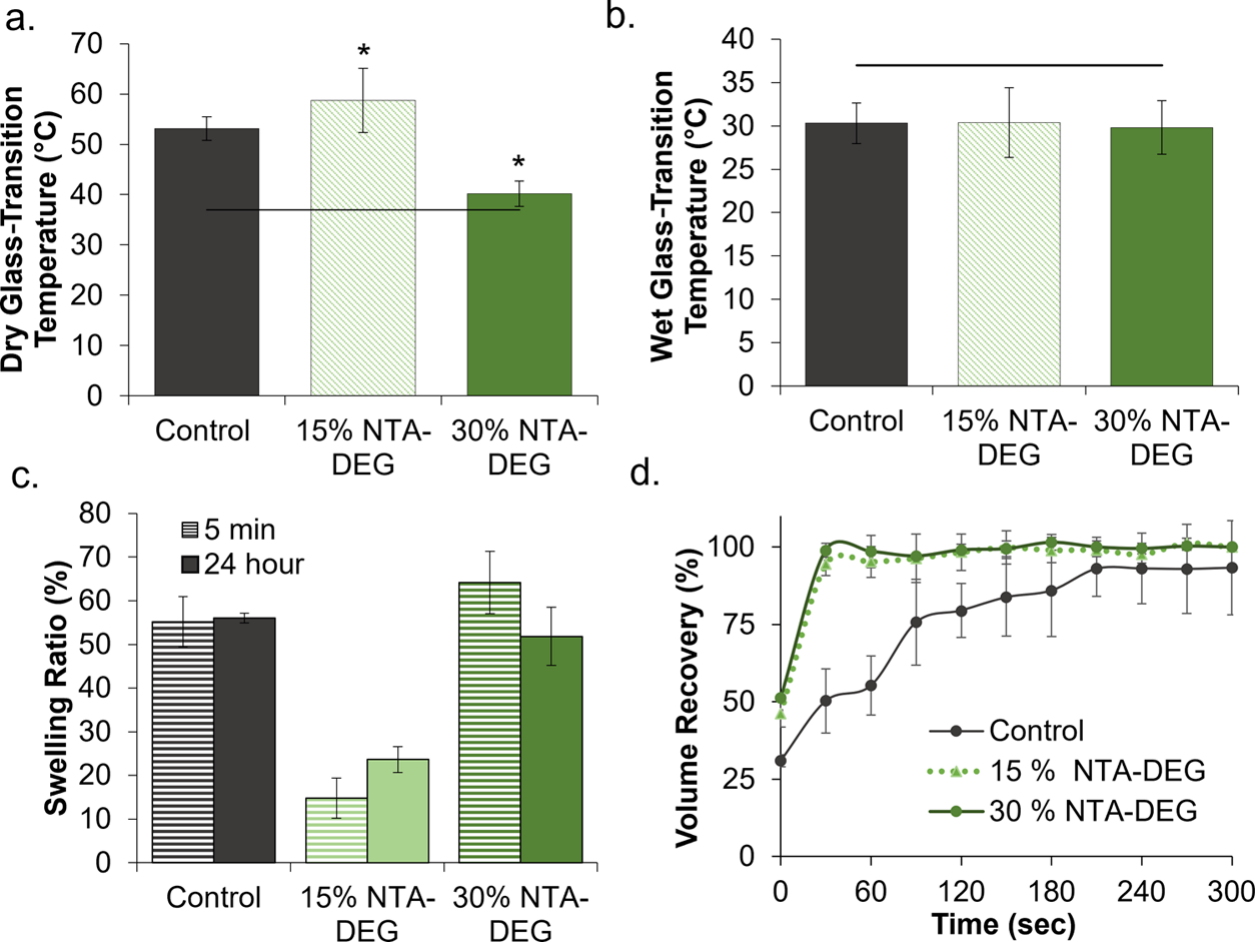
Thermal and shape memory properties of SMP foams. *T*_g_ measured via differential scanning calorimetry under (a) dry and (b) wet conditions (*n* = 3). *p < 0.05 relative to control. Horizontal lines indicate body temperature (37 °C). (c) Volumetric swelling ratios in water at 5 min and 24 h at 37 °C (*n* = 3). (d) Volume recovery profiles of foam samples in deionized water at 37 °C (*n* = 3). Average ± standard deviation displayed for all data.

The use of polyol cross-linkers with three (TEA and NTA–DEG) and four (HPED) hydroxyl groups provides an amorphous, highly cross-linked network that is indicated by the absence of melting peaks in the DSC plots. The shape memory properties are designed around the dry and wet *T*_g_’s of the system. Figure 3a shows that all foam formulations have dry *T*_g_’s over 40°C, which ensures that the biomaterials maintain their compressed secondary shape when stored in dry conditions. The wet *T*_g_’s are reduced to below 37 °C due to plasticization of the network by water, as shown in Figure 3b, which allows for the actuation of shape memory properties after exposure to water in body-temperature blood upon implantation.

### 3.3 Hydrophilicity and Shape Memory Behavior

Swelling ratios in water were calculated to provide an indication of material hydrophilicity (Figure 3c). Control foams swelled by 55% in 5 min, and the effective swelling after 24 h was 56%. Similarly, 30% NTA–DEG swelled by 64% in 5 min and 51% in 24 h, while 15% NTA–DEG swelled a significantly lower amount of 15% in 5 min and 23% in 24 h. The statistically similar swelling ratios at the 5 min and 24 h time points indicate that equilibrium swelling is reached within the first 5 min of water incubation with these foams. Shape memory properties of foams are required to ensure that they expand to their original shape after implantation and exposure to body-temperature blood. The volume recovery profiles of the foams in water at 37 °C are shown in Figure 3d. Control foams expanded back to their original shape within ∼200 s, while NTA–DEG foams reached a 100% volume recovery within ∼25 s.

### 3.4 Tensile Testing

Mechanical testing data are presented in Table 2. In general, elastic modulus and tensile strength decreased and maximum elongation increased with the addition of NTA–DEG. These trends were observed in both dry and wet/plasticized conditions. Relative differences in elastic modulus were reduced in wet conditions (15× and 41× decreases between control and 15 and 30% NTA–DEG, respectively, in dry condition vs 6× and 10× decreases in wet condition). Tensile strength was similar between samples in the wet state, and ultimate elongation differences increased in wet conditions.

**Table 2:** Tensile Properties of Shape Memory Polymer Foams in Dry and Wet Conditions^a^

	dry			wet		
sample	elastic modulus (kPa)	ultimate tensile strength (kPa)	maximum elongation (mm/m)	elastic modulus (kPa)	ultimate tensile strength (kPa)	maximum elongation (mm/m)
Control	3216 ± 1669	528 ± 230	0.17 ± 0.04	153 ± 22	53 ± 21	0.36 ± 0.19
15% NTA–DEG	217 ± 86*	69 ± 26*	0.39 ± 0.20	24 ± 5*	45 ± 20	1.73 ± 0.39
30% NTA–DEG	78 ± 33*	94 ± 7*	1.39 ± 0.60*	15 ± 3*	70 ± 24	4.53 ± 1.34*

a *n* = 3, average ± standard deviation displayed. *p < 0.05 relative to control.

### 3.5 Cell and Blood Interactions

**Figure 4:**
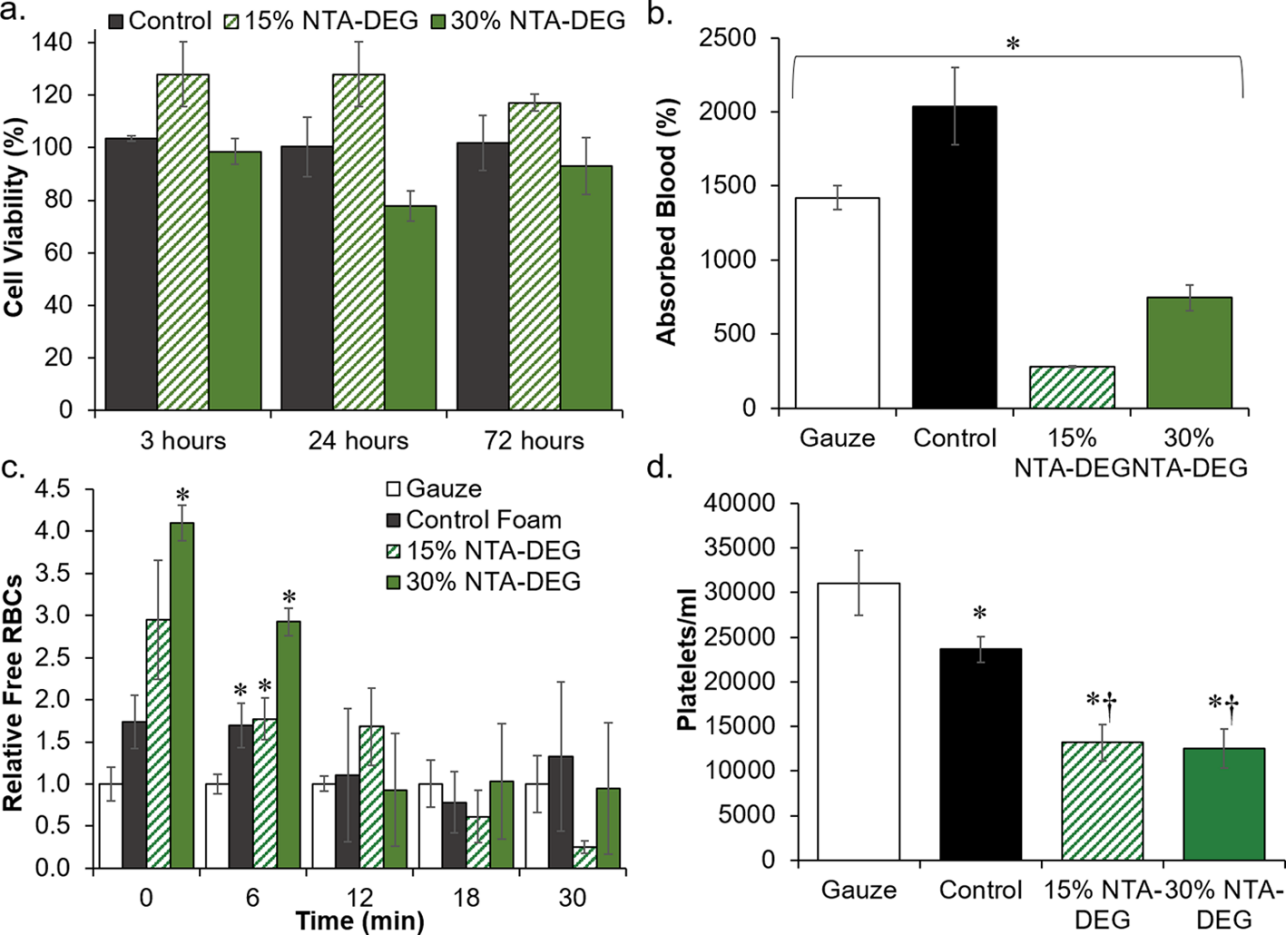
Cell and blood interactions with SMP foams. (a) 3T3 fibroblast viability over 3, 24, and 72 h (*n* = 3). (b) Blood absorption over 24 h at 37 °C (*n* = 3). *p < 0.05 between all four samples. (c) Blood clotting times in terms of free red blood cells (RBCs) relative to gauze clinical control over 30 min (*n* = 4). *p < 0.05 relative to gauze. (d) Platelet attachment concentrations measured using the LDH assay (*n* = 4). *p < 0.05 relative to gauze. †*p* < 0.05 relative to control foam. Average ± standard deviation displayed for all data.

Cell viability was >75% for all samples over 72 h (Figure 4a). Control foams absorbed significantly more blood than gauze and NTA–DEG foams (Figure 4b). The lowest blood absorption was measured in 15% NTA–DEG foams, followed by 30% NTA–DEG foams. Clotting times were measured relative to a gauze clinical control (Figure 4c). At 6 min, all SMP foams had significantly higher free red blood cells (RBCs) relative to the gauze clinical control, indicative of reduced clotting. However, by 12 min, all samples had comparable clotting levels. Images of lysates can be viewed in Figure S2 in the Supporting Information.

### 3.6 Platelet Attachment and Activation

**Figure 5:**
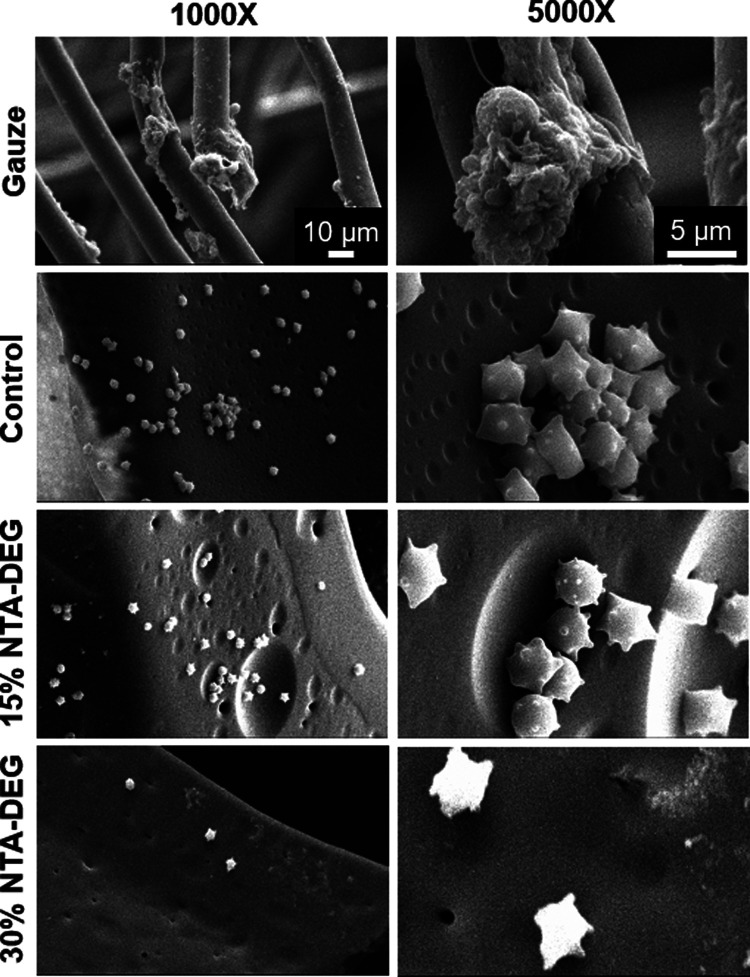
SEM micrographs of attached and activated platelets after incubation in whole porcine blood. Scale bars apply to all images in a given column.

Decreasing concentrations of PRP resulted in a linear decrease in platelet numbers that could be used to quantify the concentrations of attached platelets on samples using the LDH assay. As shown in Figure 4d, the highest levels of platelet attachment were observed on the gauze clinical control, followed by the SMP control foam. Ester-containing foams had a significantly lower concentration of attached platelets compared to that of the controls. Platelets were found on all materials in SEM images. The granules released from the cytoplasm of platelets upon activation after attachment can be visualized by the small protrusions seen in Figure 5. The gauze clinical control contained areas of thrombus formation, indicating that platelets attached, aggregated, and activated during the 30 min of whole blood incubation. All SMP formulations showed evidence of platelet attachment and activation. Aggregates were found on control and 15% NTA–DEG samples, while 30% NTA–DEG samples had lower platelet density.

### 3.7 Degradation Analysis

#### 3.7.1 Mass Loss and Physical Erosion

**Figure 6:**
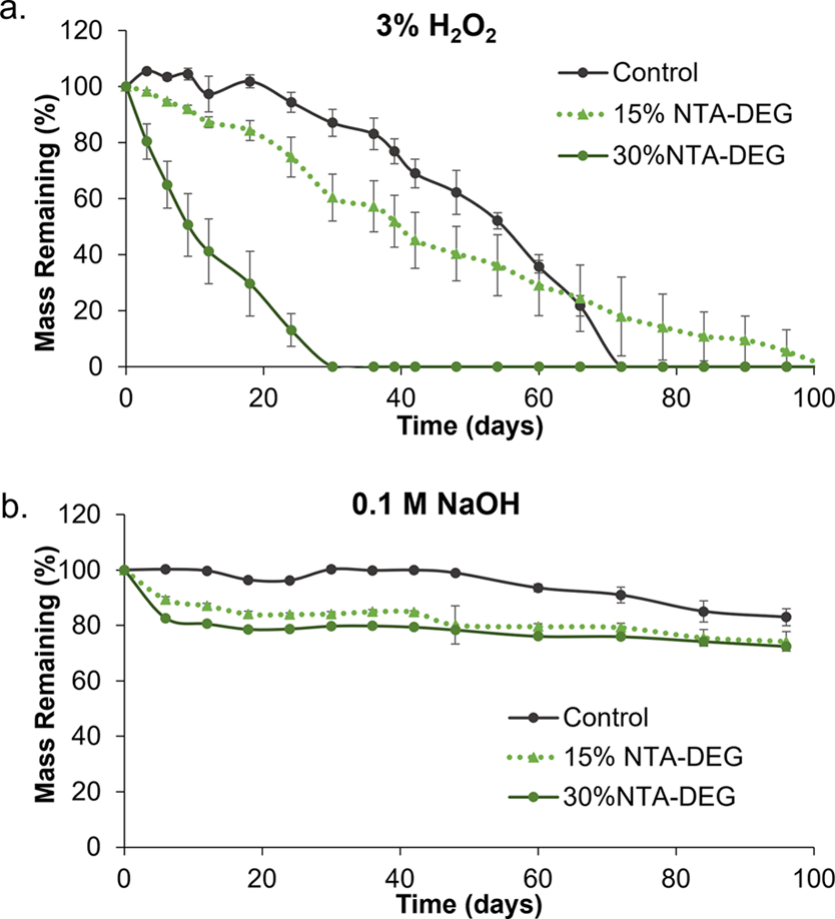
Mass loss of samples as a function of time (*n* = 5) in (a) real-time oxidative degradation media (3% H_2_O_2_) and (b) accelerated hydrolytic degradation media (0.1 M NaOH). Average ± standard deviation displayed.

**Figure 7:**
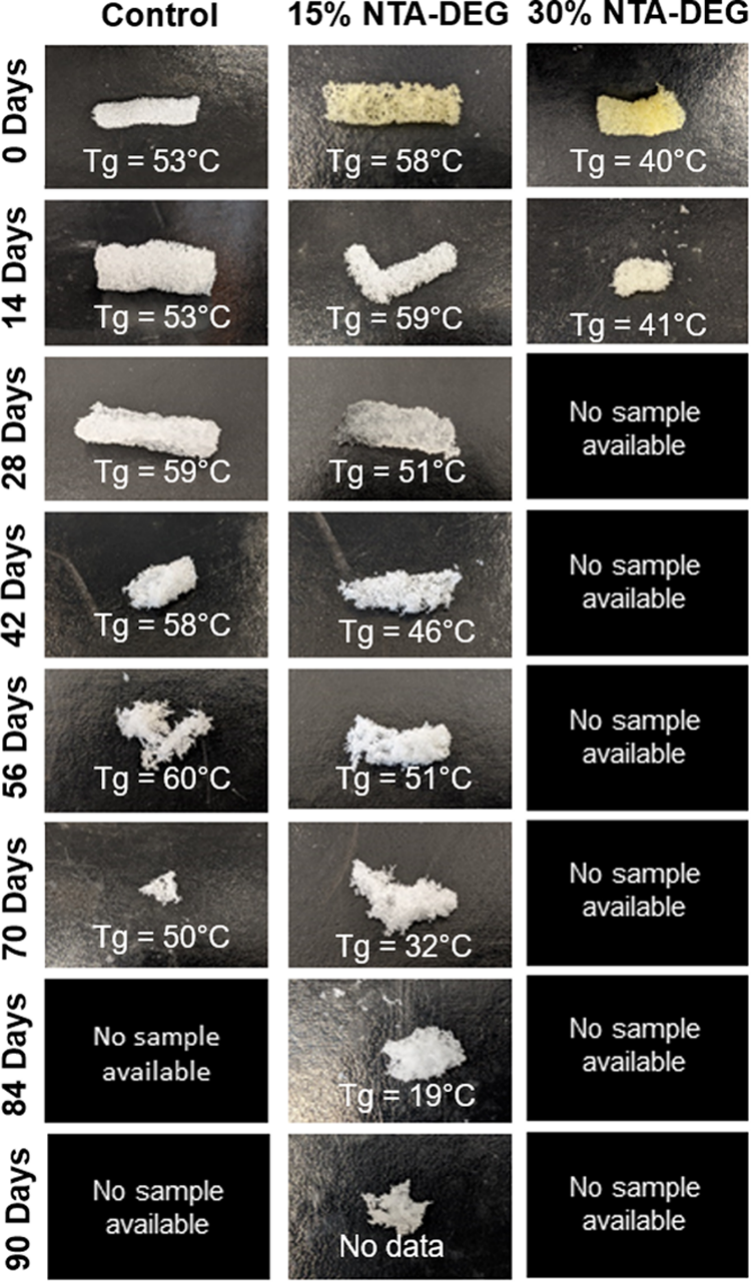
Erosion profile and glass-transition temperatures of samples during degradation in 3% H_2_O_2_. No sample was available for imaging upon almost complete degradation at ∼28 days for 30% NTA–DEG foams and at ∼84 days for controls.

Despite the inclusion of hydrolytically degradable ester linkages, NTA–DEG foams were relatively stable in 0.1 M NaOH (Figure 6b). They lost ∼20% of their mass within 1–2 weeks, after which mass loss plateaued. The remainder of the hydrolytic degradation characterization data can be found in Figures S3–S5 in the Supporting Information. In 3% H_2_O_2_, the 30% NTA–DEG foams underwent linear mass loss (*R*^2^ = 0.953) over 30 days (Figure 6a). The 15% NTA–DEG foams had a consistent, linear mass loss (*R*^2^ = 0.976) in 3% H_2_O_2_ with full degradation at 100 days. Control foams have initially slow degradation rates, and the rate increased around 42 days until 100% degradation within 72 days (*R*^2^ = 0.894). In general, NTA–DEG foams remained in a single piece throughout the degradation process, which indicates that surface erosion occurred in these samples, while control foams started to break apart into smaller pieces after ∼42 days (Figure 7).

#### 3.7.2 Thermal Analysis

Variations in *T*_g_ throughout degradation provide an indication of relative cross-link densities over time. This information can be used to determine if the materials undergo bulk degradation, where the entire network is attacked at once, or surface degradation, where the network remains relatively stable.^27−29^ Retained *T*_g_’s throughout the degradation time frame, as shown in Figure 7, indicate that the polymer networks remained fairly intact and that degradation occurred primarily on the surface of the materials. This is expected for oxidative degradation due to the high reactivity of reactive oxygen species.^30^ The 15% NTA–DEG foam *T*_g_ dropped at 70 days, indicating that bulk hydrolysis may take over as the primary degradation mechanism at this point.^29^

#### 3.7.3 Pore Morphology

**Figure 8:**
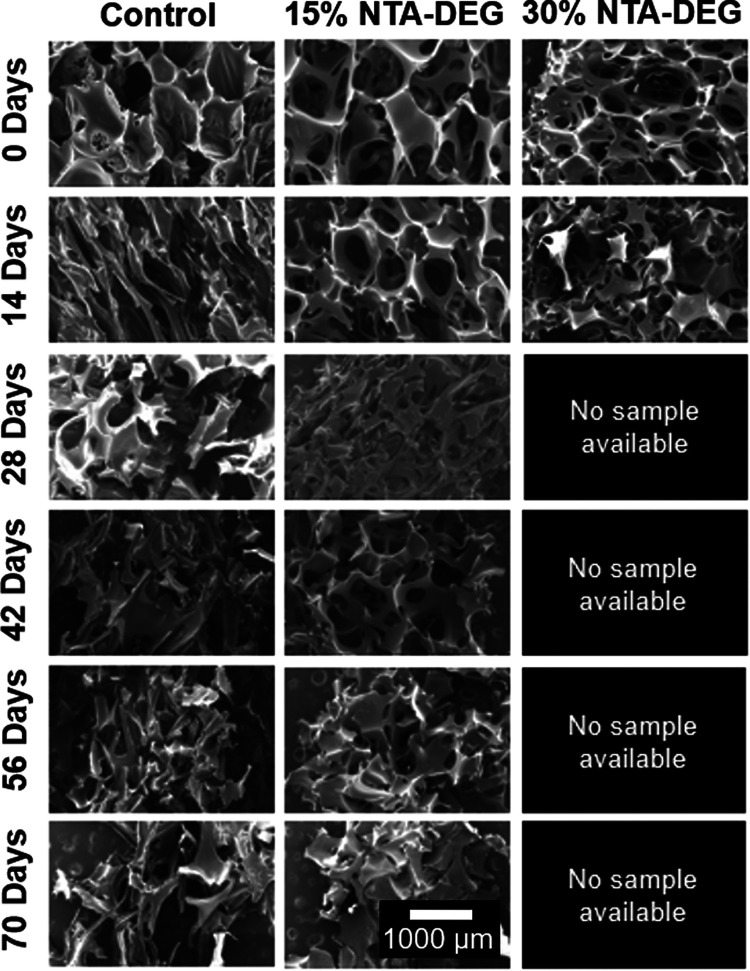
SEM micrographs of samples throughout 10 weeks of degradation in 3% H_2_O_2_. 30% NTA–DEG degraded completely by the 28 day time point. Scale bar of 1000 μm applies to all images.

Pore morphology of foams was observed every 2 weeks via SEM, as shown in Figure 8. In the case of control foams in 3% H_2_O_2_, the pores began to collapse by 14 days with significant strut breakage by 28 days. Total pore collapse at 42 days corresponds to the macroscale breaking apart of control foams shown in Figure 7. Pore morphology was the most stable in the 15% NTA–DEG foams, with visible pores and interconnects as late as 42 days, despite the increased mass loss in these samples in comparison with the controls (69 ± 5% remaining in control vs 45 ± 9% remaining in 15% NTA–DEG). The 30% NTA–DEG foams degraded too much for imaging by the 28 day time point, and evidence of significant degradation (loss of struts) can be seen by 14 days.

#### 3.7.4 Spectroscopic Analysis

**Figure 9:**
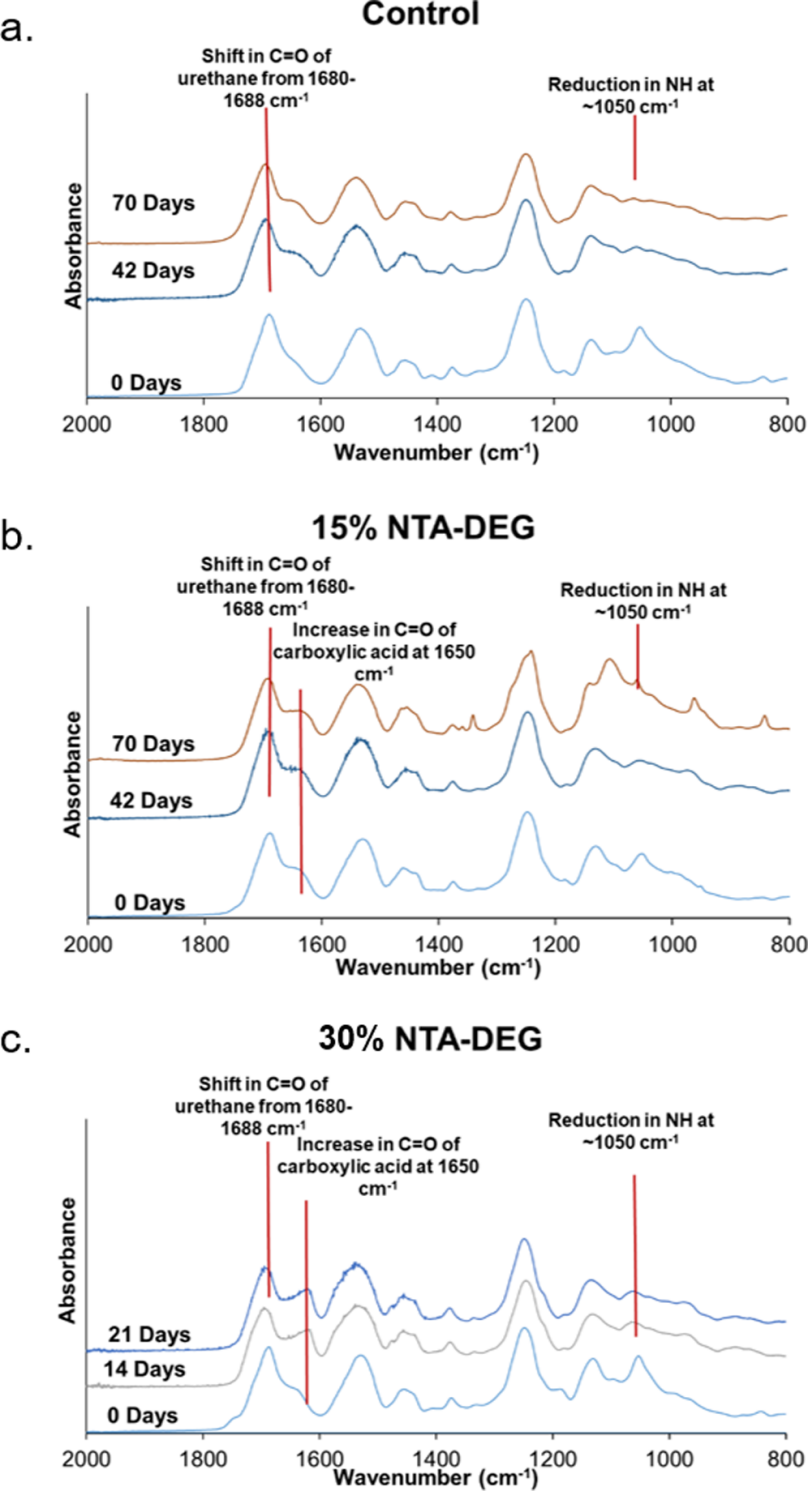
FTIR spectra of (a) control, (b) 15% NTA–DEG, and (c) 30% NTA–DEG throughout 10 weeks of degradation in 3% H_2_O_2_. 30% NTA–DEG degraded completely after the 21 day time point.

As seen in Figure 9, a shift in the carbonyl of urethane peak from 1680 to 1688 cm^–1^ and a reduction in the tertiary amine of HPED and TEA (and NTA for ester-containing foams) at 1050 cm^–1^ are an indication of oxidative degradation across all of the formulations, as previously shown.^19,23^ Among the NTA–DEG foams, an increase in the carbonyl of the carboxylic acid peak at 1650 cm^–1^ is attributed to the carboxylic acid byproduct formation during hydrolytic cleaving of ester linkages.

## 4 Discussion

### 4.1 Foam Characterization

The addition of NTA–DEG as a monomer aided in opening the pores, as demonstrated by pinholes in pore walls (Figure 2c). This increased interconnectivity between pores may be attributed to the hydrophilicity of DEG. Namely, the relatively hydrophilic DEG component increases interactions between the prepolymer/monomers and the water blowing agent and/or surfactant to aid in pore opening. Previous attempts to increase interconnectivity in SMP foams rely upon mechanical reticulation, plasma treatment, and/or use of physical blowing agents.^31,32^ Inclusion of more hydrophilic monomers presents a new method for pore opening in these materials. This interconnectivity could increase nutrient transfer throughout the biomaterial scaffold, as well as allow for tissue and blood vessel ingrowth, and it expands potential future applications for SMP foams in tissue engineering and regenerative medicine applications.^33,34^

In general, SMPs contain two main components at the molecular level, known as net points and switching segments.^35^ Net points define the permanent shape of the SMP. In this specific material system, the net points are covalent cross-links that form during synthesis (urethane linkages that form upon reaction with hydroxyls with isocyanates). Switching segments provide the mechanism for shape memory. In this specific system, the switching segments are hydrogen bonds that form between urethane linkages in between the cross-links. At temperatures above the *T*_g_, hydrogen bonds are broken between the network chains to increase flexibility, making the material elastic. This elasticity allows for deformation (radial crimping in this case) into the secondary shape. Upon cooling below the *T*_g_, new hydrogen bonds form between chains that have been rearranged during crimping, again limiting SMP flexibility and fixing the temporary shape. Upon exposure to body temperature water after shape fixation, the hydrogen bonds between the urethane linkages are interrupted by water plasticization that reduces the *T*_g_ and triggers shape recovery at a relatively lower temperature in the wet condition.

A reduction in dry *T*_g_ of 30% NTA–DEG foams can be attributed to the longer chain length and increased flexibility of NTA–DEG monomers, which theoretically reduces the overall cross-link density and network rigidity. Plasticization after exposure to water reduces the *T*_g_ in the wet state and enables actuation after exposure to body temperature (37 °C water). All foams have wet *T*_g_ below 37 °C. It was originally hypothesized that NTA–DEG foams may have lower wet *T*_g_ due to the hydrophilic DEG chains; however, wet *T*_g_’s were statistically similar for the three foam formulations. The retained wet *T*_g_ of NTA–DEG foams as compared to controls is attributed to intermolecular bonding between the dipoles of ester linkages.

Previous research on polyurethane foams shows that the materials primarily hydrogen-bond with water through the N–H groups rather than through the C=O linkages, based upon Fourier transform infrared (FTIR) spectra.^36^ Namely, when hydrogen bonds form at the N–H groups, the N–H infrared band at ∼3307 cm^–1^ increases in intensity and shifts to higher wavenumbers. When hydrogen bonding occurs via bridges between two C=O groups, the FTIR spectra show an increase in the intensity of the C=O peak at ∼1687 cm^–1^ with shifts to lower wavenumbers. In the current study, we collected FTIR spectra on foams that had been submerged in water at 50 °C for 5 min and compared those with dry foam spectra (shown in Figure S6 in the Supporting Information). The N–H peak intensities increased with general shifts to higher wavenumbers in the wet foam spectra, indicating the presence of hydrogen bonds between water and the N–H groups in the urethane linkages. Meanwhile, the C=O peaks at ∼1687 cm^–1^ were of similar intensity between the wet and dry samples, with no apparent shift in wavenumber, indicating minimal hydrogen bonding between these groups in these materials. These FTIR spectra support the hypothesis that the dipole–dipole bonds in these foams are less susceptible to water plasticization via hydrogen bonding, which would correlate with reduced effects on wet *T*_g_ in this system.

This hypothesis also correlates with the foam swelling ratios in water. Namely, there is a drop in swelling in the 15% NTA–DEG foams that may be attributed to the dipole–dipole bonds with reduced water access. With an increase in the NTA–DEG content to 30%, the hydrophilicity of DEG overcomes these restrictions to increase water interactions with the network. The faster volume recovery observed with NTA–DEG foams is likely due to the open pore structure, which enables faster water penetration and shape recovery from the compressed form. This property could be beneficial for hemostatic dressing use, as it would allow for faster wound filling after implantation.^37^

Based on the theoretically reduced cross-link density and open pore structure in NTA–DEG foams, the dry mechanical property trends (decreased elastic modulus and tensile strength and increased ultimate elongation with NTA–DEG incorporation) were generally expected. Some of these differences could also be attributed to the reduced density of 30% NTA–DEG foams. The smaller differences in modulus and strength between NTA–DEG foams and controls in the wet state are again attributed to secondary intermolecular forces in the samples. The carbonyl linkages of the ester groups interact via secondary dipole interactions that are less affected by water in comparison with the hydrogen bonds between urethane linkages. Thus, plasticization does not affect the NTA–DEG foam flexibility to the same extent as that of control foams. While the wet mechanical properties are more similar between formulations, it may be beneficial to tune the stiffness of NTA–DEG foams in future studies by increasing HPED content or decreasing diisocyanate monomer chain length with butane diisocyanate in place of HDI.

### 4.2 Biological Characterization

All of the foams retained high cytocompatibility (>75%) over 72 h, which meets the ISO 10993 standard for cytocompatibility.^38^ Future studies will focus on measuring cytocompatibility of degradation products and in vivo host response during degradation to provide a better understanding of the material biocompatibility. The absorbed blood amounts generally correlate with swelling ratios in water, with the control foam having the highest volume of absorbed blood and 15% NTA–DEG having the lowest volume. The decrease in blood absorption in the 30% NTA–DEG foam relative to the control foam could be attributed to the open pore structure, which likely reduced the amount of retained (anticoagulated) blood after removal.

Clotting times were measured relative to gauze. SMP foams all had slower clotting times with increased free RBCs at 0 and 6 min. However, by 12 min, all samples had fully clotted with no differences between free RBC levels. In general, the 30% NTA–DEG foam appeared to clot the slowest of the tested formulations. This trend was also seen in the platelet attachment numbers, where gauze had the highest number of platelets, followed by the control foam. The NTA–DEG foams had the lowest platelet numbers. The platelet images correlate with these results based on evidence of more advanced thrombus formation on the gauze sample. All SMP formulations promoted platelet attachment and activation, demonstrated by the protrusions on individual platelets. Control and 15% NTA–DEG foams had areas with platelet aggregates, a precursor to thrombus formation. The 30% NTA–DEG foams had the lowest levels of imaged platelet numbers and aggregates.

Interestingly, in our preliminary in vivo experiments in a porcine liver injury,^39^ treatment with control foams slightly reduced blood loss and significantly increased animal survival in comparison with gauze treatment. Thus, based upon comparisons between gauze and control SMP foams, the in vivo clotting process is more complex than we are able to replicate with these initial in vitro studies and likely requires further investigation in less static conditions. However, it does appear that the modification with degradable NTA–DEG reduces the clotting capabilities of SMP foams. One of the main benefits of the SMP foam system is its synthetic tunability. We have parallel work that involves incorporation of antimicrobial phenolic acids into SMP foams to reduce infection risks.^40^ In addition to their antimicrobial properties, phenolic acids demonstrate procoagulant activity.^41,42^ Future work will focus on incorporating procoagulant species, such as phenolic acids, into the NTA–DEG foams to increase their clotting capabilities while maintaining the desired degradation profiles, open pore structures, and flexible mechanical properties.

### 4.3 Degradation Profiles

Control SMP foams have excellent hydrolytic stability. Despite the incorporation of hydrolytically labile ester linkages in NTA–DEG foams, they were very stable in accelerated hydrolytic degradation media after an initial drop in mass by ∼20% at the first time point. We hypothesize that this stability could be due to (i) relative hydrophobicity around the ester linkages to reduce water penetration into the network and (ii) initial degradation of only ester linkages and loss of NTA from the polymer, after which the remainder of the network was stable in 0.1 M NaOH. Based on prior studies showing that control foams degrade via oxidation of tertiary amines in HPED and TEA monomers, we hypothesized that NTA–DEG foams may degrade more quickly in oxidative conditions.^19,23^ As the network degrades oxidatively, it becomes more hydrophilic, enabling increased water access to the hydrolytically degradable ester linkages to promote hydrolysis. Thus, degradation characterization was focused on samples in oxidative media (3% H_2_O_2_). Control foams had an initially slow degradation rate with an apparent increase in the rate at ∼42 days. The change in degradation rate of control foams is consistent with previous SMP foam degradation studies and corresponds with the observed erosion profile.^19,23^ This result could be attributed to the relative brittleness of control foams (higher elastic modulus) that causes bulk erosion upon exposure to external forces during the characterization process. Namely, the foams are washed, dried, and weighed every 3–7 days. Increased brittleness can cause samples to break apart over time of repeated external force application. The nonlinear mass loss rates and differences in erosion profiles would likely affect tissue regeneration and load transfer in vivo as well and make estimation of in vivo degradation rates more complex.^43−45^

The NTA–DEG monomer was designed to provide multiple potential degradation points. NTA contains a tertiary amine similar to HPED and TEA, which has been previously shown to break down into carboxylic acid and ammonia byproducts.^46^ The carboxylic acid groups locally decrease pH within the scaffold, which can accelerate hydrolysis of the ester linkages between NTA and DEG. Finally, DEG contains ether linkages, which (i) enhance hydrophilicity adjacent to the ester linkage to increase water access and (ii) may also degrade oxidatively. Ether oxidation produces carboxylic acids, alcohols, and aldehydes, and the carboxylic acids can further catalyze ester hydrolysis.^15^ During the ether degradation process, there is a possibility that the carbon radicals can cross-link with each other to form cross-links. While cross-linking was not visible on the FTIR spectra (would be indicated by branched ether peak at ∼1172 cm^–1^), it is possible that ether cross-linking occurred simultaneously to oxidative and hydrolytic degradation in 15% NTA–DEG foams to provide a more linear degradation profile and reduce bulk erosion.

The degradation rate of NTA–DEG foams can be easily tuned with variations in the NTA–DEG content. We hypothesize that degradation rates could be further controlled with other monomer variables, such as diisocyanate length, monomer hydrophobicity, and polyol functionality. These foams have linear in vitro mass loss rates that, if replicated in vivo, could be highly beneficial for graded load transfer from scaffolds to tissues during healing, particularly when considering the highly interconnected pores. While the focus of the current work is on hemorrhage control, these foams provide a potential platform for tissue engineering scaffolds in future work.

## 5 Conclusions

This study reveals that incorporation of ester linkages using the new NTA–DEG monomer can increase the degradation rate of polyurethane SMP foams to clinically relevant time frames of 4–8 weeks^24^ while maintaining desired thermal properties. Namely, a dry *T*_g_ above 40 °C ensures that the foams can be stored in their secondary shape, and a wet *T*_g_ below body temperature enables expansion to the primary shape after implantation. The new monomer also imparted other potentially valuable foam properties, including interconnects between the pores that may aid in tissue regeneration, very rapid volume recovery within 30 s to aid in hemostatic dressing delivery, and increased flexibility to potentially reduce tears or premature breakdown of scaffolds during delivery or healing. Incorporation of NTA–DEG reduced the clotting capabilities of SMP foams; thus, future studies with these foams will include incorporation of procoagulant species into the tunable material system. Beyond hemorrhage control, these foams provide a platform for future development into other regenerative medicine applications where scaffold degradation is desired.

## Supporting Information Available

FTIR and 1H NMR spectrum of the synthesized NTA–DEG ester; representative images of lysates from clotting assay; glass transition temperatures and erosion profile of samples throughout degradation in 0.1 M NaOH; SEM micrographs for samples throughout degradation in 0.1 M NaOH; FTIR spectra of samples throughout degradation in 0.1 M NaOH; and FTIR spectra of dry samples and water interactions with polyurethane foams (PDF)
