# Supplementary material for: Shape Memory Polymer Foams with Tunable Degradation Profiles
Source: ACS Appl Bio Mater. 2021 Aug 11;4(9):6769–79. doi: 10.1021/acsabm.1c00516 (PMC8456454; doi:10.1021/acsabm.1c00516)
Supplement: Supplementary file 1 — mt1c00516_si_001.pdf [file mt1c00516_si_001.pdf]

Shape Memory Polymer Foams with Tunable Degradation Profiles

Anand Utpal Vakil[a], *Natalie Marie Petryk*[a], *Ellen Shepherd*[a], *Henry T. Beaman*[a], *Priya S. Ganesh*[a], *Katheryn S. Dong*[a], *and Mary Beth B. Monroe*[a]*

[a]Department of Biomedical and Chemical Engineering, Syracuse Biomaterials Institute, and BioInspired Syracuse: Institute for Material and Living Systems, Syracuse University, Syracuse, NY 13244.

*mbmonroe@syr.edu

KEYWORDS: shape memory polymers, polyurethanes, oxidation, degradation, foams

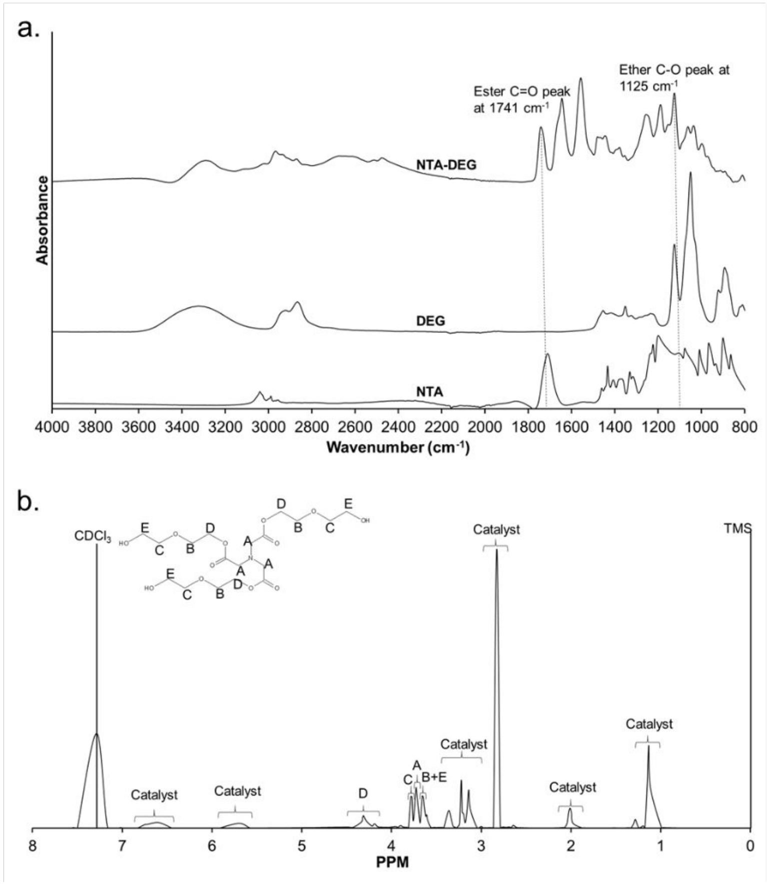

Figure S1. (a) Fourier transform infrared (FTIR) and (b) nuclear magnetic resonance (NMR) spectra of nitrilotriacetic acid-diethylene glycol (NTA-DEG) monomer.

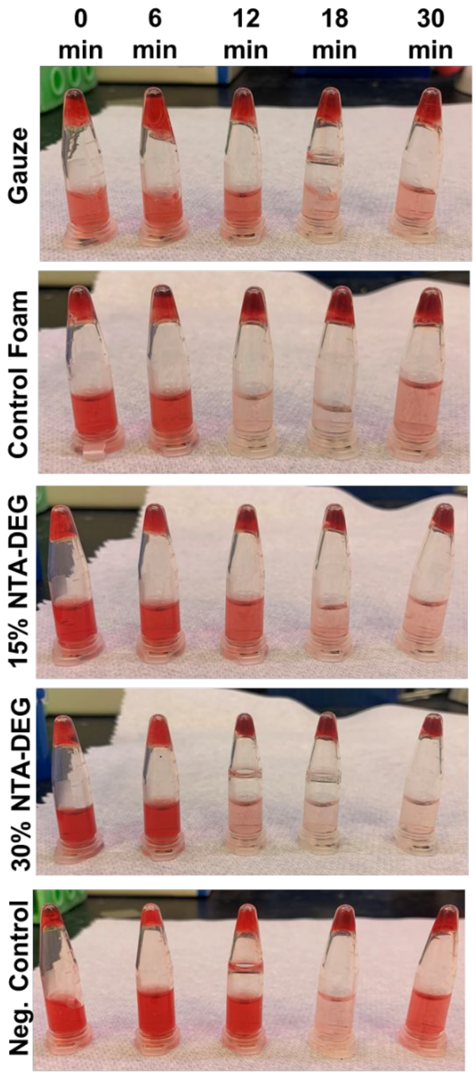

Figure S2. Representative images of lysates from clotting assay. Neg. control: Empty tube.

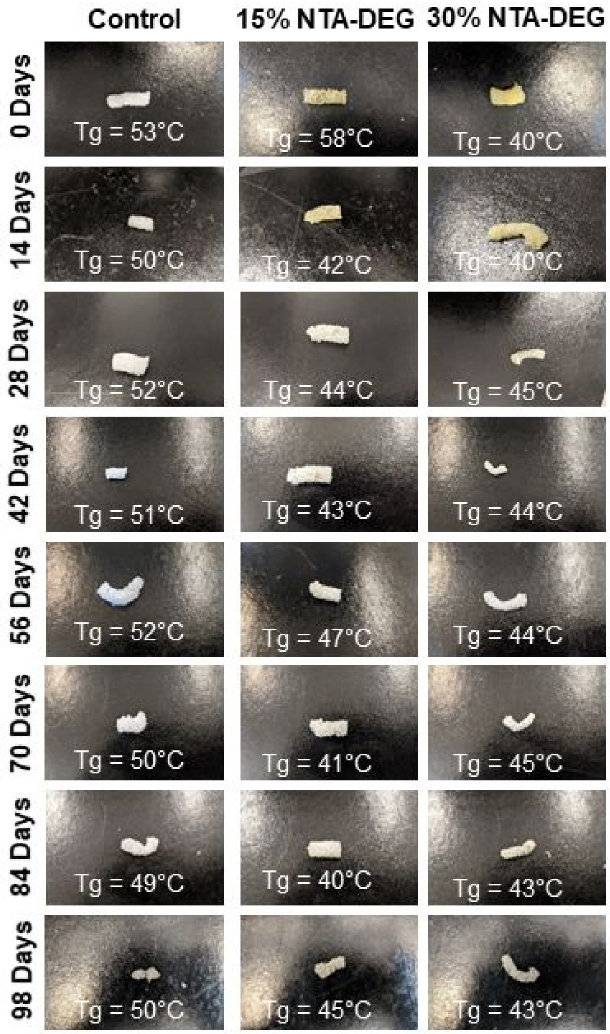

Figure S3. Erosion profile and glass transition temperatures of samples during degradation in 0.1 M NaOH.

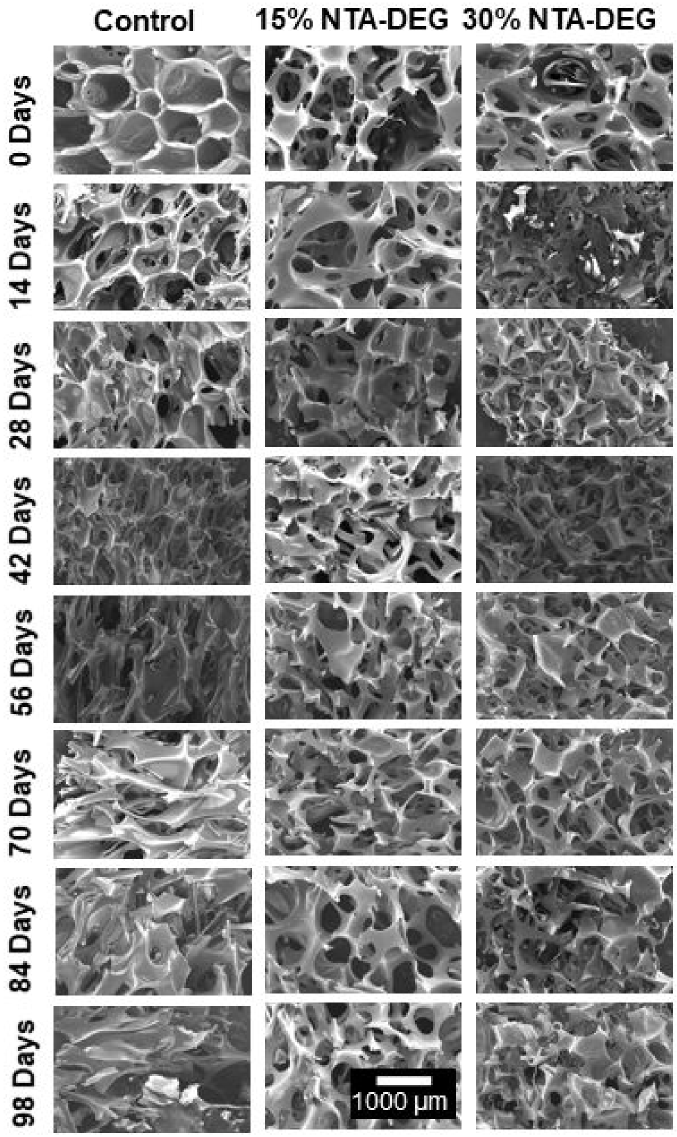

Figure S4. SEM micrographs of samples throughout degradation in 0.1M NaOH. Scale bar of 1000 µm applies to all images.

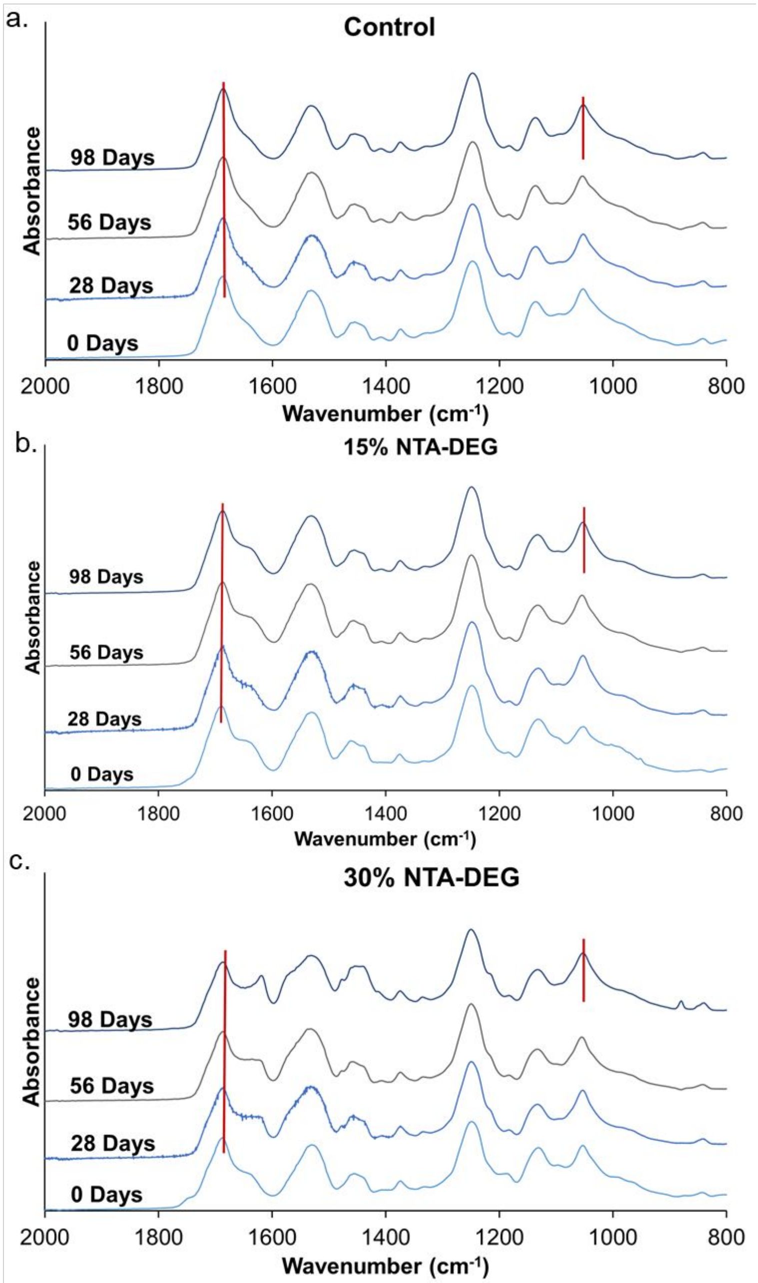

Figure S5. FTIR spectra of (a) Control, (b) 15% NTA-DEG, and (c) 30% NTA-DEG throughout degradation in 0.1M NaOH.

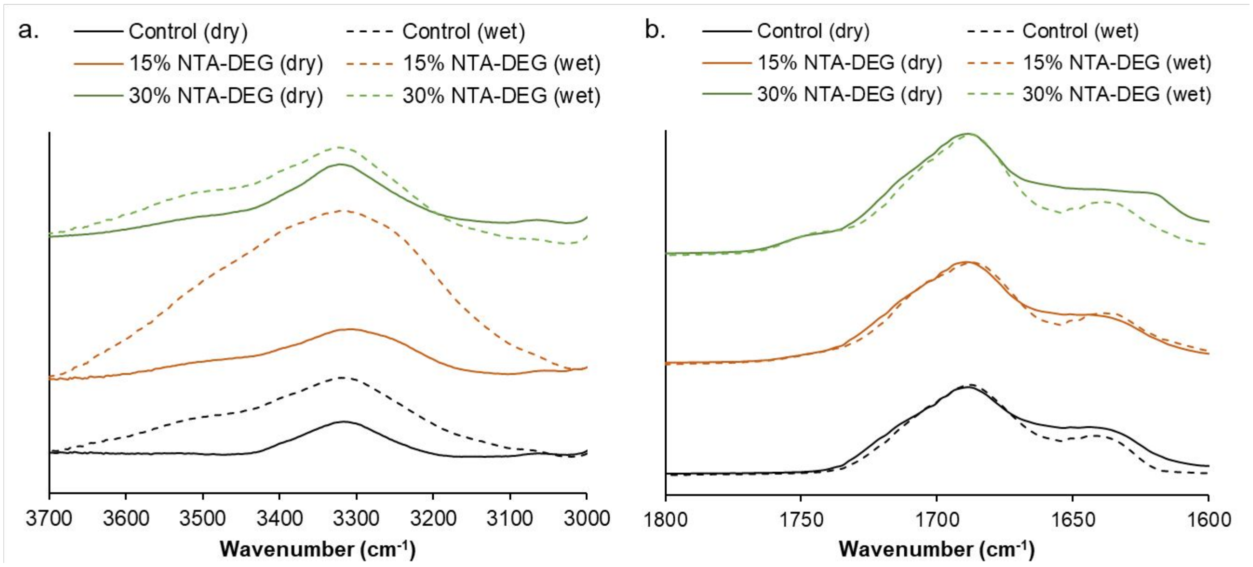

Figure S6. FTIR spectra of foam samples in dry and wet state. (a) The N-H peak at ~3308 cm^{-1} and (b) the C=O peak at ~1687 cm^{-1}.
